# New serum soluble factors predicting inflammatory and non-inflammatory disability worsening in multiple sclerosis

**DOI:** 10.3389/fimmu.2025.1729500

**Published:** 2025-12-05

**Authors:** Alexander Rodero-Romero, María Domínguez-Mozo, Enric Monreal, José M. García-Domínguez, Noelia Villarrubia, José Ignacio Fernández-Velasco, Manuel Comabella, Susana Sainz de la Maza, Haydee Goicochea Briceño, Juan Luís Chico-García, María Ángel García-Martínez, Fernando Rodriguez-Jorge, José Luis Veiga-Gonzalez, Raquel Sainz-Amo, María Luisa Martínez Ginés, Xavier Montalban, Jaime Masjuan, Lucienne Costa-Frossard, Roberto Álvarez-Lafuente, Luisa María Villar

**Affiliations:** 1 Department of Immunology, Hospital Universitario Ramón y Cajal, Red Española de Esclerosis Múltiple (REEM), Red de Enfermedades Inflamatorias (REI), Instituto de Salud Carlos III (ISCIII), Instituto Ramón y Cajal de Investigación Sanitaria, Madrid, Spain; 2 Grupo Investigación de Factores Ambientales en Enfermedades Degenerativas, Instituto de Investigación Sanitaria del Hospital Clínico San Carlos, Madrid, Spain; 3 Department of Neurology, Hospital Universitario Ramón y Cajal, Red Española de Esclerosis Múltiple (REEM), Red de Enfermedades Inflamatorias (REI), Instituto de Salud Carlos III (ISCIII), Instituto Ramón y Cajal de Investigación Sanitaria, Madrid, Spain; 4 Department of Neurology, Hospital General Universitario Gregorio Marañón, Madrid, Spain; 5 Servei de Neurologia, Centre d’Esclerosi Múltiple de Catalunya, Institut de Recerca Vall d’Hebron, Hospital Universitari Vall d’Hebron, Universitat Autònoma de Barcelona, Barcelona, Spain; 6 Center for Networked Biomedical Research on Neurodegenerative Diseases (CIBERNED) - Instituto de Salud Carlos III (ISCIII), Madrid, Spain

**Keywords:** multiple sclerosis, serum biomarkers, serum proteomics, neuroimmunology, demyelinated diseases

## Abstract

**Introduction:**

Serum neurofilament light chains (sNfL) and glial fibrillary acidic protein (sGFAP) associate respectively with acute inflammation and smoldering disease in relapsing-remitting multiple sclerosis (MS) patients. We explored the proteomic profile associated with the different combinations of low or high levels of sNfL and sGFAP to explore immune mechanisms involved in different MS outcomes.

**Methods:**

Multicenter cross-sectional study including 253 treatment-naïve PwMS, and 180 healthy controls (HCs). sNfL and sGFAP levels were measured via SIMOA and patients were classified according to their levels into four groups: NLGL (normal sNfL and sGFAP), NHGL (high sNfL/normal sGFAP), NHGH (high sNfL and sGFAP), and NLGH (normal sNfL/high sGFAP). Serum proteomics were measured using the Olink™ 48 Cytokine panel. Results were analyzed by Kruskal-Wallis tests with Dunn’s *post hoc* analysis and further corrected by the False Discovery Rate analysis. Q-values below 0.05 were considered as significant.

**Results:**

Compared with HC, patients with low sGFAP concentrations exhibited decreased levels of granulocyte-macrophage colony stimulating factor (GM-CSF), a cytokine inducing innate cell activation q=0.009 for NLGL and q=0.002 for NHGL groups). Conversely, patients with high sGFAP values showed a remarkable decrease of epidermal growth factor (EGF), a cytokine involved in remyelination and axon repair produced by resting astroglia (q<0.0001 for NHGH and NLGH respectively). Additionally, NLGH patients exhibited pronounced decreases in soluble mediators essential for adaptive immune activation, including FLT3LG, TNFSF12, and TNF-α, compared to HC. They also showed broad reductions in chemokines involved in leukocyte recruitment as CCL3, CCL7, and CCL4. The most important decrease was found in CCL2, a chemokine that attracts monocytes and memory T cells to the CNS. NLGH patients showed lower values than HC (q<0.0001), NLGL (q=0.02), NHGL (q=0.02), and NHGH (q=0.03). These findings indicated that NLGH patients experience a reduction of the inflammatory response in the peripheral blood and a decreased immune cell attraction to the central nervous system combined with a lower ability of tissue repair.

**Discussion:**

We identified new molecules implied in the different pathological mechanisms occurring in patients with MS. These biomarkers could improve patient stratification, outcome prediction, and treatment optimization.

## 1 Introduction

Recent advances in serum biomarkers in multiple sclerosis (MS) contribute to establishing patient prognosis and monitoring treatment response (1–6). High serum neurofilament light chain (sNfL) levels are linked to neuroinflammation and identify patients at risk of relapse-associated worsening (RAW) (2–7). sNfL reflects acute axonal injury driven by focal inflammatory activity. Their levels associate with active demyelinating lesions and acute inflammation (8). Additionally, elevated serum glial fibrillary acidic protein (sGFAP) levels reflect astrogliosis and have been associated with a higher risk of progression independent of relapse activity (PIRA) (5–7). It reflects chronic, compartmentalized CNS inflammation, mediated by astrocytes, which contribute to ongoing tissue damage (9) and suggests a disbalance in astrocyte pro-inflammatory (A1) and neuroprotective (A2) states (10, 11). sGFAP provides, in junction with sNfL insights into the different mechanisms contributing to MS pathology.

We used these biomarkers to further investigate soluble factors present in the serum of patients with relapsing–remitting MS (PwMS) classified according to their sNfL and sGFAP values. Doing so allowed us to better understand the pathological processes underlying patients at risk of inflammatory and non-inflammatory worsening. This approach is useful for identifying novel biomarkers that refine patient stratification and may ultimately guide more precise therapeutic strategies for MS.

Soluble immune-related factors contribute to key processes such as immune cell activation, blood–brain barrier permeability, tissue damage and repair (12–15). Immune-related factors in MS have been mainly studied in the cerebrospinal fluid (CSF), since the effector mechanisms in MS are mostly confined to the central nervous system (CNS) (16–19). However, because inflammation in MS is believed to originate outside the CNS (12, 13), exploring the contribution of circulating immune-related molecules becomes essential to improving our understanding of the diverse pathological mechanisms driving the disease. Although several serum molecules (19, 20) and cellular markers (21) have been associated with MS, the overall understanding remains incomplete.

Recent advances in high-sensitivity proteomic technologies are creating new opportunities to investigate the immune landscape in MS (22, 23). In this context, we aimed to explore differences in soluble biomarkers between healthy controls (HCs) and PwMS, applying this technology and stratifying patients according to their sNfL and sGFAP values.

## 2 Methods

### 2.1 Study design and participants

This multicenter, cross-sectional study included individuals with PwMS and HCs. We adhered to the STrengthening the Reporting of OBservational studies in Epidemiology (STROBE).

The inclusion criteria for PwMS were as follows: an MS diagnosis according to the 2017 McDonald criteria at sample extraction or during follow-up (24), a relapsing remitting MS form, an age between 18 and 65 years, an absence of other neurodegenerative or autoimmune diseases, a lack of treatment with any disease-modifying therapies (DMTs), a disease duration of less than 1 year, and a lack of corticosteroids treatment for at least 2 months prior to sample collection.

The inclusion criteria for HCs were as follows: an age between 18 and 65 years, no history or evidence of any neurodegenerative or autoimmune disease, no alcohol abuse or illicit drug use, and no current treatment with any immunosuppressive or chemotherapeutic agents. HCs were hospital employees who volunteered to participate by providing blood samples. Each HC underwent an interview conducted by a neurologist and showed normal sNfL values; however, no physical examination was performed.

Patients and HCs were recruited between January 2022 and December 2024 at three university hospitals in Spain. We included 253 PwMS recruited consecutively and 180 HCs. No participants were excluded from the final analysis.

### 2.2 Samples

We collected serum samples by drawing 10 ml of blood into dry tubes. The blood was then centrifuged at 3000×g for 5 minutes. After centrifugation, the serum was divided into 250 µl aliquots and stored at −80 °C until analysis. All of the samples were processed using standardized protocols by trained personnel, which reduced measurement bias.

### 2.3 sNfL and sGFAP quantification

sNfL and sGFAP were measured by single molecule array technique (SIMOA) using the SIMOA NF-light™ Advantage Plus Kit and the SIMOA™ GFAP Advantage Plus Kit, respectively (Quanterix, Billerica, MA, USA), on an HD-X instrument (Quanterix), according to the manufacturer’s instructions. sNfL concentrations were normalized using z-scores adjusted for age and body mass index (BMI), as previously described (4). PwMS were classified into NLGL (normal sNfL and sGFAP), NHGL (high sNfL/normal sGFAP), NHGH (high sNfL and sGFAP) and NLGH (normal sNfL/high sGFAP) according to recent literature (6, 7). Cut-off values were defined as previously reported (25). A z-score ≥1.5 was considered indicative of elevated sNfL levels. High sGFAP levels were defined as concentrations above 140 pg/ml for individuals under 55 years of age and above 280 pg/ml for individuals aged 55 years or older.

### 2.4 Quantification of serum soluble factors

Serum soluble factors were quantified using the Olink™ Target 48 Cytokine panel on an Olink™ Signature Q100 instrument (Thermo Fisher, MA, USA). This platform allows for the simultaneous analysis of 45 immune-related factors, including cytokines, chemokines, and other pro- and anti-inflammatory proteins. **Supplementary Table S1** provides a complete list of the soluble factors included in the panel.

The Olink^®^ Target 48 Cytokine assay uses Proximity Extension Assay (PEA) technology with a two-step normalization and strict quality control framework to ensure precision and cross-run comparability. Each well contains internal controls to monitor the immunoreaction, extension, and qPCR phases. Normalization involves correcting analyte Cq values by the Extension Control (ΔCq), then by the median Calibrator ΔCq across plates (ΔΔCq), and finally adjusting by a bridging factor to align different kit lots. External controls (calibrator, sample, and negative controls) were used to assess precision, accuracy, and background. Quality control criteria required intra- and inter-assay CVs < 30%, control deviations within ±0.3 values, and exclusion of samples failing these limits.

Missing data were generally very low, below 1% in 42 out of the 45 proteins analyzed. Only three proteins (MMP1, TSLP, and IL33) exhibited missing data levels above 3% (see **Supplementary Table S2**). No assumptions were made for missing values.

### 2.5 Statistical analysis

Categorical variables are presented as numbers and percentages [n (%)]. Differences between groups were analyzed using the chi-squared test and Fisher’s exact test.

We used the Mann-Whitney and Kruskal-Wallis tests to compare continuous variables. For the Kruskal-Wallis test, we conducted *post-hoc* analyses between groups using Dunn’s Multiple Comparison Test. All statistical tests were two-tailed, and a p-value of less than 0.05 was considered to indicate statistical significance. P-values were corrected for multiple comparisons using the False Discovery Rate (FDR) method. After correction q values lower than 0.05 were considered as significant. Spearman test correlation was used to assess the relationships between soluble factors and clinical parameters. Volcano plots were generated to visualize the differential distribution between groups.

The statistical analyses were conducted using GraphPad Prism 9.0 (GraphPad Prism Inc., San Diego, CA, USA) and R software (version 4.3.2; R Foundation for Statistical Computing, Vienna, Austria).

### 2.6 Ethics statement

The study was approved by the Ethics Committee of the Ramon y Cajal University Hospital. At the meeting held on 09/26/2023 record 459, it was decided to issue the positive report corresponding to the study. All participants provided written informed consent to participate.

### 2.7 Data availability

The original data will be accessible to any researcher in the field for 3 years upon request to the corresponding author.

## 3 Results

### 3.1 Characterization of the patient cohort

We included 253 PwMS (74% women, median age 37.9 years [IQR: 28.64–45.95]) and 180 HCs (72% women, median age 38.1 years [IQR: 28.9–48.2]). No statistical differences in sex or age were found between PwMS and HCs.

We classified PwMS according to their sNfL and sGFAP levels as NLGL (93 patients), NHGL (n=71), NHGH (n=55) and NLGH (n=34). Values of sNfL and sGFAP in every patient group and in HCs are shown in **Supplementary Table S3**.

Demographic and baseline clinical characteristics of the different groups of patients are summarized in **Table 1**. The only significant differences were observed in the number of gadolinium (Gd+) enhancing lesions, that were higher in the NHGH group compared with the NLGL (p=0.007) and NLGH (p=0.02) groups.

**Table 1:** Demographic and clinical data of the cohorts included in the study.

Demographic and clinical data	NLGL(n=93)	NHGL (n=71)	NHGH (n=55)	NLGH (n=34)
Age (years)	36.4 [29.3-47.4]	37 [27-49.2]	38 [31.2-46]	40.2 [25.5-45.1]
Sex female	67 (72.1)	47 (69.1)	43 (78)	26 (76.5)
Disease duration (days)	71 [12-189]	60 [17-153]	82 [10-192]	97 [52.3-198.5]
EDSS at baseline	1.5 [1-2]	1.5 [1-2]	1.5 [1.5-2]	1.5 [1.5-2]
Topography of first relapse				
Optic nerve	22 (23.7)	11 (15.4)	9 (16.3)	6 (17.7)
Brainstem	20 (21.5)	18 (25.4)	12 (21.8)	8 (23.5)
Spinal cord	36 (38.7)	28 (39.4)	24 (43.6)	13 (38.2)
Multifocal	4 (4.3)	3 (4.2)	2 (3.6)	2 (5.9)
Others	11 (11.8)	11 (15.5)	8 (14.7)	5 (14.7)
T2 lesions at baseline				
0	5 (5.4)	0 (0)	1 (2.8)	2 (5.9)
1-3	18 (19.3)	9 (12.7)	4 (7.3)	4 (11.7)
4-9	23 (24.7)	21 (29.6)	11 (20)	10 (29.4)
10-50	41 (44.1)	36 (50.7)	30 (54.5)	14 (41.2)
>50	6 (6.5)	5 (7)	9 (16.4)	4 (11.8)
Gd+ enhancing lesions	**0 [0-1]**	1 [0-1]	1 [0-3] ¶** θ*	**0 [0-1]**
IgG oligoclonal bands	87 (93.5)	66 (92.9)	50 (90.9)	31 (91.2)

Continuous variables are expressed as medians and 25-75% interquartile ranges (25-75% IQRs). Differences between groups were examined using the Kruskal–Wallis multiple comparisons test and, with Dunn’s *post hoc* test. Significant comparisons are highlighted in bold. EDSS: Expanded Disability Status Scale; Gd: Gadolinium; IgG: Immunoglobulin G; IQR: Interquartile Range; NHGH: Patients with high sNfL Z-scores and sGFAP; NHGL: Patients with high sNfL Z-scores and normal sGFAP values; NLGH: Patients with low sNfL Z-scores and high GFAP levels; NLGL: Patients with low sNfL Z-scores and GFAP values. θ: difference with the NLGH group; PwMS: Patients with MS; ¶: difference with the NLGL group;*: p<0.05 and **: p<0.01 obtained with Dunn’s test. In all cases, significant p values in Dunn’s test implied p values < 0.05 in the associated Kruskal–Wallis test.

### 3.2 Association of soluble factors with the different MS profiles

We next explored the differences between the serum values of 45 soluble factors within the four groups of PwMS and with the HCs group. Results including Dunn’s corrected p values are shown in **Supplementary Table S4**. Differences were further corrected using The FDR test and variables showing significant differences are shown in **Table 2**.

**Table 2:** Serum soluble factors showing significant differences in our study.

Soluble factors (pg/ml)	HCs (n=180)	NLGL (n=93)	NHGL (n=71)	NHGH (n=55)	NLGH (n=34)
Factors associated with inflammation					
FLT3LG	**109 [93.4-130]**	99.4 [78.9-120]	99.5 [83.4-124]	102 [81.4-134	87 [65-111] ⱡ***
GM-CSF	**0.15 [0.1-0.2]**	0.1[0.08-0.2] ⱡ**	0.1[0.07-0.15] ⱡ**	0.1 [0.08-0.2]	0.1 [0.06-0.4]
IL-4	**0.05 [0.02-0.08]**	0.03 [0.02-0.06] ⱡ*	0.03 [0.01-0.05] ⱡ*	0.02 [0.01-0.06] ⱡ**	0.02 [0.01-0.04] ⱡⱡ**
IL13	**0.5 [0.3-1.1]**	0.3 [0.2-0.7] ⱡ**	0.3 [0.2-0.7] ⱡ*	0.3 [0.2-0.7] ⱡ**	0.2 [0.1-0.8] ⱡ**
TNF-α	**19.5 [16.7-23.5]**	18.4 [15.1-23.2]	17.6 [14.3-2.8]	17.3 [13.6-22.7]	15 [12.7-20] ⱡ**
TNFSF12	**843 [726-1015]**	812 [671-960]	805 [672-951]	790 [646-945]	734 [531-855] ⱡ**
Chemokines					
CCL2	595 [477-769] θ****	532 [397-754] θ*	554 [405-714] θ*	550 [383-716] θ*	**397 [209-536]**
CCL3	**9.4 [6.9-22.2]**	8.3 [5.8-13.1]	8.5 [5.9-16.1]	7.6 [5.8-10.3]	6.2 [5.4-9.7] ⱡ**
CCL4	**135 [95.3-183]**	128 [95-182]	128 [93.8-170]	119 [94.4-172]	99 [70.2-131] ⱡ**
CCL7	**1.1 [0.8-1.8]**	0.9 [0.7-1.4]	0.69 [0.6-1.3]	0.9 [0.6-1.4]	0.7 [0.5-1.1] ⱡ*
Factors involved in repair processes					
EGF	**604 [359-750]**	383[209-614] ⱡ**	**512 [304-727]**	343 [165-539] ⱡ**** ϒ*	311 [127-182] ⱡ**** ϒ**

Data are presented as median [25–75% IQR] in pg/ml, with statistically significant differences highlighted in bold. Q-values were calculated from Dunn’s test p-values after adjustment with the False Discovery Rate (FDR) method. HCs: healthy controls; IQR: interquartile range; NHGH: patients with high sNfL Z-scores and sGFAP values; NHGL: patients with high sNfL Z-scores and normal sGFAP values; NLGH patients with normal sNfL Z-scores and high GFAP levels; NLGL: patients with normal sNfL Z-scores and GFAP values. ⱡ: difference with the HCs group; θ: difference with the NLGH group; ϒ: difference with the NHGL group; * q<0.05, ** q<0.01, *** q<0.001, and **** q<0.0001 obtained from Dunn’s *post-hoc* test and adjusted using the False Discovery Rate correction.

Our analysis revealed distinct patterns in inflammation-related soluble factors, chemokines, and repair-associated molecules among the PwMS and the HCs groups.

First, we observed decreased values of TH2 cytokines in all groups of PwMS patients compared with the HCs. IL-4 levels were higher in the HCs compared with the NLGL group (q=0.02), the NHGL group (q=0.04), the NHGH group (q=0.008), and the NLGH group (q=0.006). Similarly, the HCs exhibited higher IL-13 values than the NLGL group (q=0.003), the NHGL group (q=0.04), the NHGH group (q=0.002), and the NLGH group (q=0.002) (**Supplementary Figure S1**). We did not find any other common differences across all MS groups compared with the HCs.

**Figure 1:**
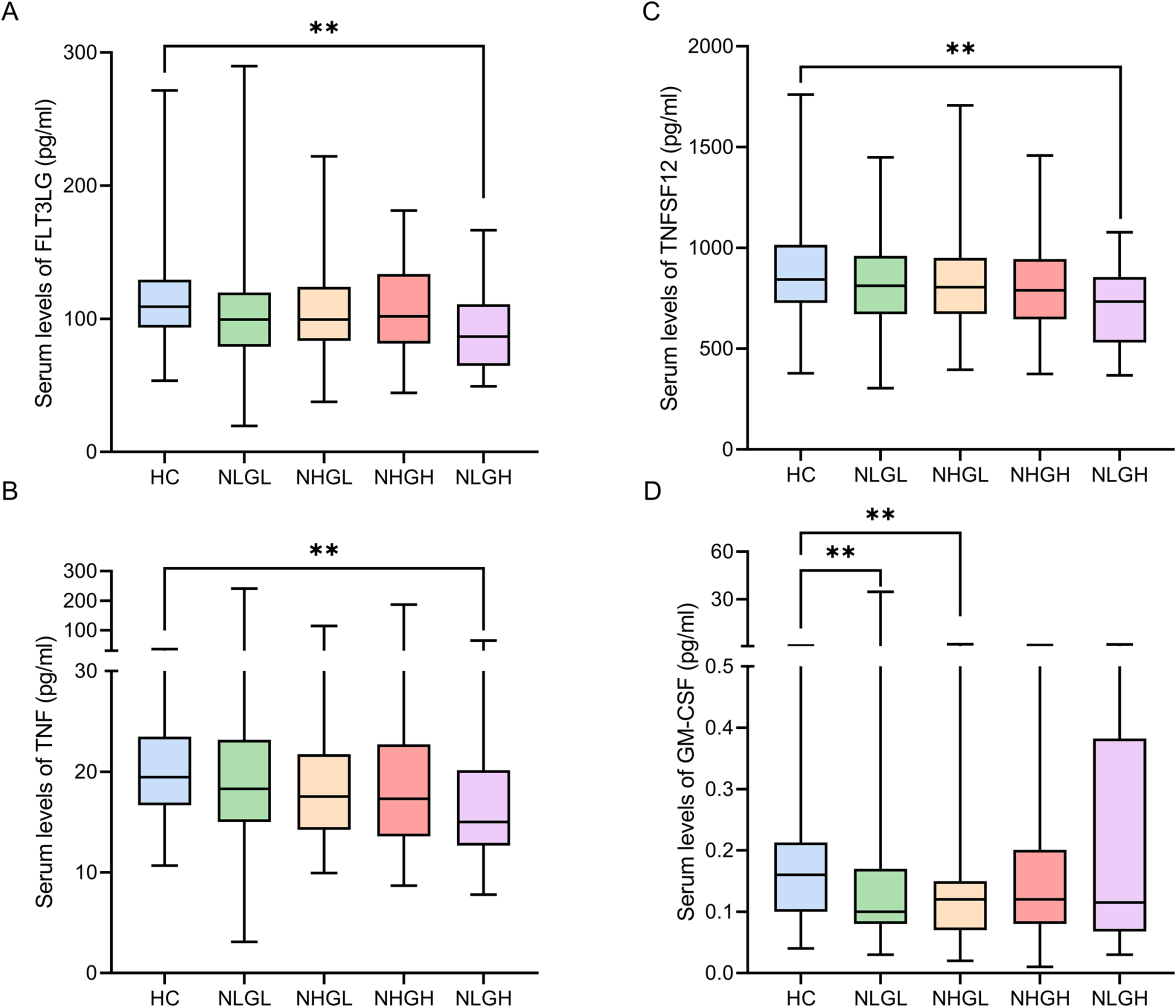
Serum levels (pg/ml) of inflammation-related soluble factors in PwMS groups and HCs: FLT3LG **(A)**, TNF-α **(B)**, TNFSF12 **(C)**, and GM-CSF **(D)**. FLT3LG: Fms-related tyrosine kinase 3 ligand; GM-CSF: Granulocyte-macrophage colony-stimulating factor; HC: Healthy controls; NHGL: Patients with high sNfL z-scores and normal sGFAP values; NLGH: Patients with low sNfL z-scores and high GFAP levels; NLGL: Patients with low sNfL z-scores and GFAP values; PwMS: patients with MS; TNF-α: Tumor necrosis factor alpha; TNFSF12: Tumor necrosis factor ligand superfamily member 12; **q < 0.01, obtained from Dunn’s *post-hoc* test and adjusted using the false discovery rate method.

However, when comparing controls with different MS groups, we identified distinct differences within these groups. Notably, GM-CSF levels were reduced in the NLGL (q=0.009) and NHGL (q=0.002) groups compared to HC. Of note, we found clear differences that were mainly restricted to NLGH group. Compared with the HCs, individuals in the NLGH group exhibited decreased levels of FLT3LG (q=0.003), a cytokine essential for dendritic cell immune response; TNFSF12 (q=0.004), a regulator of tissue remodeling and B cell activity; and TNF-α (q=0.004), a key mediator of Th1 immune response (**Figures 1A–D**).

**Figure 2:**
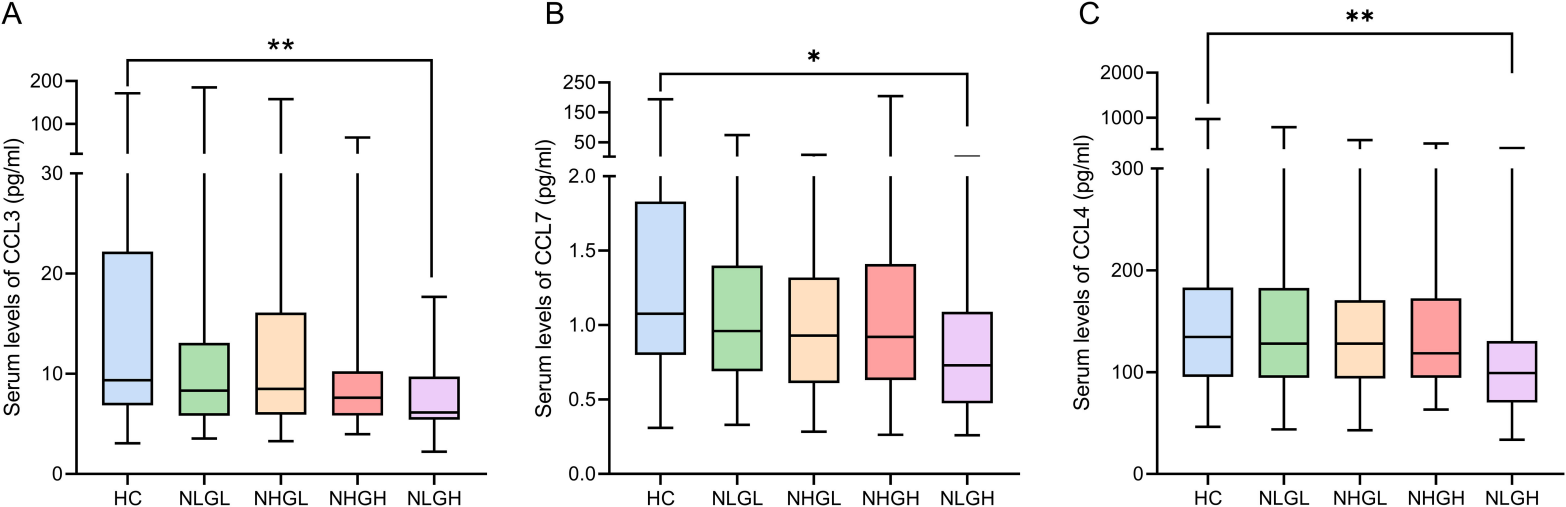
Serum levels (pg/mL) of chemokines in PwMS groups and HC: CCL3 **(A)**, CCL7 **(B)**, and CCL4 **(C)**. CCL3: C-C motif chemokine 3; CCL4: C-C motif chemokine 4; CCL7: C-C motif chemokine 7; HCs: Healthy controls; NHGL: Patients with high sNfL z-scores and normal sGFAP values; NLGH: Patients with low sNfL z-scores and high GFAP levels; NLGL: Patients with low sNfL z-scores and GFAP values; PwMS: patients with multiple sclerosis; *q<0.05; **q<0.01 obtained from Dunn’s *post-hoc* test and adjusted using the false discovery rate correction.

**Figure 3:**
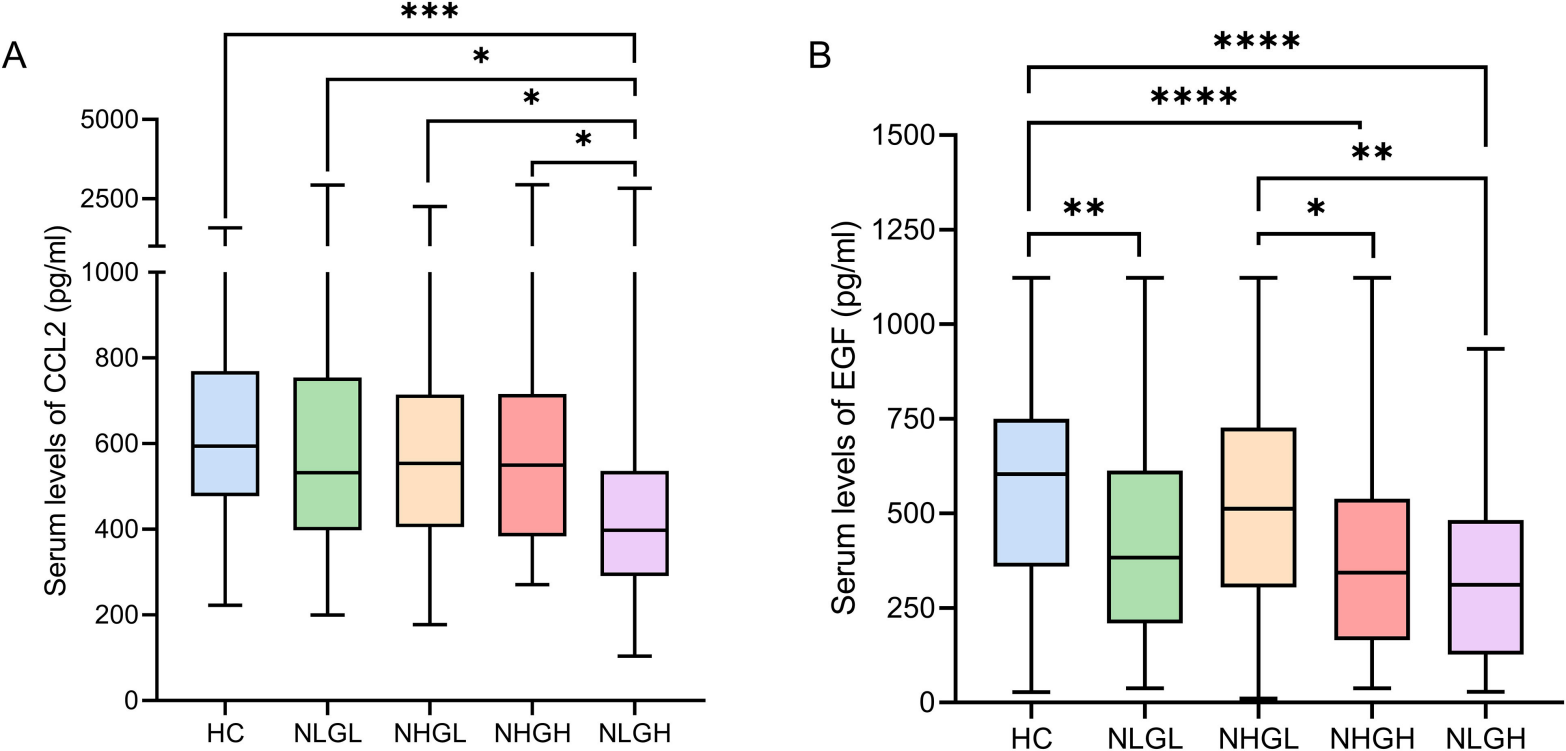
Serum levels (pg/ml) of the chemokine CCL2 **(A)** and the repair factor EGF **(B)** among PwMS groups and HCs. CCL2: C-C motif chemokine 2; EGF: Epidermal growth factor; HCs: Healthy controls; NHGL: Patients with high sNfL z-scores and normal sGFAP values; NLGH: Patients with low sNfL z-scores and high GFAP levels; NLGL: Patients with low sNfL z-scores and GFAP values; PwMS: patients with MS; VEGFA: Vascular endothelial growth factor A; *q<0.05, **q<0.01, ***q<0.001, and ****q<0.0001 obtained from Dunn’s *post-hoc* test and adjusted using the False Discovery Rate method.

Additionally, patients in the NLGH group showed a clear decrease in various chemokines involved in immune cell recruitment compared with the HCs and other patients groups. Levels of CCL3 (q=0.009), CCL7 (q=0.02), and CCL4 (q=0.007) (**Figures 2A–C**). CCL2, showed the most clear differences, since its values in NLGH group were lower than those of all other groups (q<0.0001 vs HC, q=0.02 vs NLGL, q=0.04 vs NHGL, and q=0.04 vs NHGH, **Figure 3A**).

Finally, we analyzed soluble factors related to tissue repair; we found lower epidermal growth factor (EGF) levels in the NLGL (q=0.009), NHGH (q<0.0001), and NLGH (q<0.0001) groups compared with the HCs. In addition, the NHGH (q=0.03) and NLGH (q=0.009) groups exhibited decreased values compared with the NHGL group (**Figure 3B**).

**Figure 4:**
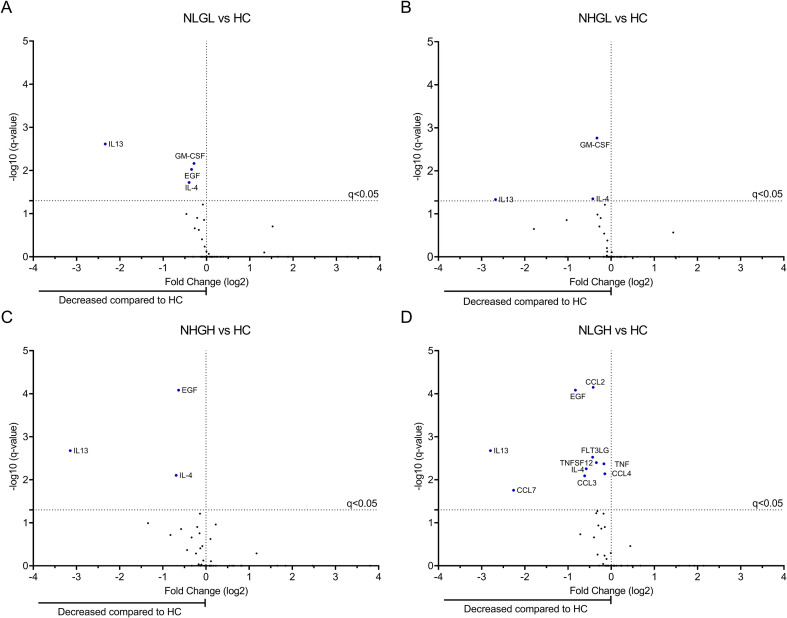
Volcano plots showing the differential levels of soluble factors between groups. Comparisons were performed between **(A)** NLGL vs HC, **(B)** NHGL vs HC, **(C)** NHGH vs HC, and **(D)** NLGH vs HC. The x-axis represents the log_2_ fold change in soluble factor levels between groups, while the y-axis represents the –log_10_ of the False Discovery Rate adjusted p-values (q-values). HCs: Healthy controls; NHGL: Patients with high sNfL z-scores and normal sGFAP values; NLGH: Patients with low sNfL z-scores and high GFAP levels; NLGL: Patients with low sNfL z-scores and GFAP values.

To further visualize differences in the soluble factors between all groups, we performed Volcano plots. Differences with HC are shown in **Figure 4**.

Finally, we explored the correlation between key soluble factors and clinical markers including EDSS after the first relapse, sNfL and sGFAP. We found a moderate negative correlation between EGF and sGFAP (r=-0.32 and p<0.0001) thus suggesting that astroglial inflammation associates with lower ability for tissue repair.

## 4 Discussion

MS is a heterogeneous disease that ranges from a relatively benign course to high disability progression associated or independent of inflammatory activity (2, 6, 12). Different approaches have been made to find immunological factors associated with MS course. In this line, two serum soluble factors, sNfL and sGFAP, have been found to be closely associated with inflammation and neurodegeneration in MS (4–7). The combination of this factors helps to identify MS patients at risk of disease progression, associated or independent of inflammation (6).

We classified our patients according to the values of these biomarkers and explored the differences between inflammatory and non-inflammatory disability progression by studying various immune-related factors using the OLINK™ proteomics platform.

Our analysis revealed altered soluble factors in all PwMS as well as in specific patient groups. Across all of the MS patient groups, we noted lower values of serum IL-4 and IL-13 compared with the HCs. This finding suggests an inhibition of the TH2 response in MS; however, we did not find any increase in Th1 and TH17 cytokines associated with proinflammatory responses previously observed in the disease (21, 26–29). This finding is somewhat surprising, as PwMS individuals exhibit high percentages of T and B cells producing TNF-α, IFN-ϒ, or IL-17 cytokines in peripheral blood (21). However, this result can be explained by the need for reactivation of these cells to produce high levels of inflammatory cytokines, as the increase of their levels in CSF could suggest (16, 30).

Of note, the main differences in this study were observed when analyzed particular MS groups. Compared with HCs, NLGH patients showed decreased values of TNF-α, a potent inflammatory mediator, which contributes to immune cell infiltration into the CNS (26); of serum TNFSF12, involved in tissue remodeling and B cell activity, which is elevated in CSF of MS patients and associated with disease activity (31); and of FLT3LG, a key factor for the development and survival of dendritic cells which supports the adaptive immune responses (32). This may be consistent with the compartmentalization of the immune response and high risk of PIRA experienced by these patients (2, 6, 7, 33). We found that NLGH patients also showed a decrease of several chemokines related to the recruitment of immune cells. These individuals showed a decrease in CCL3, CCL7 and CCL4 compared with HC. These chemokines play key roles in recruitment of monocytes, T lymphocytes, and natural killer (NK) cells to sites of inflammation (34, 35). However, the most important results were observed with CCL2, a key chemokine in recruiting monocytes, memory T cells, and dendritic cells to the CNS (36). Previous studies have described a modest decrease of CCL2 in MS patients (37), but we observed that it was restrained to NLGH group, with values remarkably lower compared to all other MS groups and HCs. In junction, these data could be consistent with the compartmentalization of the immune response in these patients within the CNS with a clear reduction of leukocyte activation in the periphery and a drastic inhibition of their migration, which may contribute to the onset of PIRA (2, 6, 33, 38).

On the other hand, the two groups of patients with low sGFAP values (NLGL and NHGL) exhibited reduced serum levels of GM-CSF, a cytokine that plays a central role in amplifying astrocyte and microglial activation (39, 40). These findings could explain the low astrocyte activation experienced by these patients and their low risk of PIRA (33).

Finally, we observed changes in EGF, a soluble factor related to tissue repair. It supports oligodendrocyte proliferation, differentiation, and survival, which is essential for CNS remyelination (41), and for axonal repair (42). A reduction in EGF levels was previously found in the CSF and serum of MS patients, and its administration was shown to improve an experimental model of the disease (43). We observed a decrease in EGF in three of the MS groups. The NLGL patients exhibited a modest decrease compared with the HCs. This finding indicates that despite favorable clinical outcomes, the reduction in the capacity of these patients for CNS repair may render them susceptible to disease progression over time. On the other hand, the NHGL patients maintained relatively unchanged EGF levels, suggesting a retained reparative capacity. That situation may be associated with a lower risk of disease progression, regardless of inflammatory activity (29). Remarkably, the lower levels of EGF observed in NLGH and NHGH patients can associate with the higher risk of progression shown by these two groups of patients (4–7, 33). In fact, the negative association found between sGFAP and EGF in this study supports the data previously found in MS experimental models showing that astroglia activation reduces its reparative function (44, 45). In MS these data are consistent with the lack of repair as a cofactor with innate cell activation inducing a high risk of PIRA (7, 33). These findings highlight EGF as a putative biomarker of reparative potential and a therapeutic target to enhance remyelination, even in mild cases of MS.

The combination of EGF and CCL2 may differentiate patients at risk of PIRA who are susceptible of responding or not to drugs targeting the adaptive immune response in MS.

The study has some limitations. Its cross-sectional design does not allow longitudinal assessment of biomarker dynamics. Since a brain MRI was not performed in HC group the possibility of a radiologically isolated syndrome could not be completely excluded. Exploring CSF samples would further clarify changes associated with compartmentalized inflammation. Potential confounders, such as vitamin D levels and HbA1c, which could influence the biomarker levels were not explored. Finally, the NLGH subgroup had a limited sample size due to the low proportion of these patients at disease onset.

In summary, this study shows that stratifying MS patients by sNfL and sGFAP levels reveals distinct immune and reparative mechanisms underlying disease heterogeneity and identifies new biomarkers that might contribute to improving treatment selection and monitoring in MS. Further studies are warranted to explore the clinical utility of these molecules.

## Data Availability

The original data will be accessible to any researcher in the field for 3 years upon request to the corresponding author.
