# Correction: New serum soluble factors predicting inflammatory and non-inflammatory disability worsening in multiple sclerosis

**DOI:** 10.3389/fimmu.2025.1774415

**Published:** 2026-01-09

**Authors:** Frontiers Production Office

**Affiliations:** Frontiers Media SA, Lausanne, Switzerland

**Keywords:** multiple sclerosis, serum biomarkers, serum proteomics, neuroimmunology, demyelinated diseases

The affiliation “Independent, Medellin, Columbia” for reviewer Ana Londono was erroneously given as “Independent, Cocos Islands”.

The original version of this article has been updated.

