# Supplementary material for: New serum soluble factors predicting inflammatory and non-inflammatory disability worsening in multiple sclerosis
Source: Front Immunol. 2025 Dec 5;16:1729500. doi: 10.3389/fimmu.2025.1729500 (PMC12714895; doi:10.3389/fimmu.2025.1729500)
Supplement: Supplementary file 1 [file Table1.doc]

Supplementary Material

**Supplementary Table 1.** Abbreviations and name of cytokines, chemokines and other immunological factors measured in serum using Olink™ Target 48 Cytokine on an Olink signature 100 device

| Abbreviation | Name of the molecule |
| --- | --- |
| CCL2 | C-C motif chemokine 2 |
| CCL3 | C-C motif chemokine 3 |
| CCL4 | C-C motif chemokine 4 |
| CCL7 | C-C motif chemokine 7 |
| CCL8 | C-C motif chemokine 8 |
| CCL11 | Eotaxin |
| CCL13 | C-C motif chemokine 13 |
| CCL19 | C-C motif chemokine 19 |
| CSF1 | Macrophage colony-stimulating factor 1 |
| CSF3 | Granulocyte colony-stimulating factor |
| CXCL8 | Interleukin-8 |
| CXCL9 | C-X-C motif chemokine 9 |
| CXCL10 | C-X-C motif chemokine 10 |
| CXCL11 | C-X-C motif chemokine 11 |
| CXCL12 | Stomal cell-derived factor 1 |
| EGF | Pro-epidermal growth factor |
| FLT3LG | Fms-related tyrosine kinase 3 ligand |
| GM-CSF | Granulocyte-macrophage colony-stimulating factor |
| HGF | Hepatocyte growth factor |
| IFNG | Interferon gamma |
| IL1B | Interleukin-1 beta |
| IL2 | Interleukin-2 |
| IL4 | Interleukin-4 |
| IL6 | Interleukin-6 |
| IL7 | Interleukin-7 |
| IL10 | Interleukin-10 |
| IL13 | Interleukin-13 |
| IL15 | Interleukin-15 |
| IL17A | Interleukin-17A |
| IL17C | Interleukin-17C |
| IL17F | Interleukin-17F |
| IL18 | Interleukin-18 |
| IL27 | Interleukin-27 |
| IL33 | Interleukin-33 |
| LTA | Lymphotoxin-alpha |
| MMP1 | Interstitial collagenase |
| MMP12 | Macrophage metalloelastase |
| OLR1 | Oxidized low-density lipoprotein receptor 1 |
| OSM | Oncostatin-M |
| TGFA | Protransforming growth factor alpha |
| TNF-α | Tumor necrosis factor alpha |
| TNFSF10 | Tumor necrosis factor ligand superfamily member 10 |
| TNFSF12 | Tumor necrosis factor ligand superfamily member 12 |
| TSLP | Thymic stromal lymphopoietin |
| VEGFA | Vascular endothelial growth factor A. |

**Supplementary Table 2**. Missing data of serum levels of immune-related soluble factors.

| Soluble factors (pg/ml) | Individuals (n=433) |
| --- | --- |
| CSF1 | 0 (0%) |
| CSF3 | 1 (0.25%) |
| FLT3LG | 0 (0%) |
| GM-CSF | 2 (0.5%) |
| IFNG | 0 (0%) |
| IL1B | 0 (0%) |
| IL2 | 0 (0%) |
| IL-4 | 0 (0%) |
| IL6 | 1 (0.25%) |
| IL7 | 0 (0%) |
| IL10 | 0 (0%) |
| IL13 | 0 (0%) |
| IL15 | 0 (0%) |
| IL17A | 1 (0.25%) |
| IL17C | 0 (0%) |
| IL17F | 0 (0%) |
| IL18 | 2 (0.5%) |
| IL27 | 0 (0%) |
| IL33 | 60 (14%) |
| LTA | 0 (0%) |
| MMP1 | 13 (3.1%) |
| MMP12 | 0 (0%) |
| OLR1 | 0 (0%) |
| OSM | 0 (0%) |
| TNF | 0 (0%) |
| TNFSF10 | 0 (0%) |
| TNFSF12 | 0 (0%) |
| TSLP | 32 (7.5%) |
| CCL2 | 3 (0.75%) |
| CCL3 | 0 (0%) |
| CCL4 | 0 (0%) |
| CCL7 | 0 (0%) |
| CCL8 | 0 (0%) |
| CCL13 | 0 (0%) |
| CCL11 | 0 (0%) |
| CCL19 | 0 (0%) |
| CXCL8 | 0 (0%) |
| CXCL9 | 2 (0.5%) |
| CXCL10 | 0 (0%) |
| CXCL11 | 4 (1%) |
| CXCL12 | 0 (0%) |
| EGF | 4 (1%) |
| HGF | 0 (0%) |
| TGFA | 0 (0%) |
| VEGFA | 0 (0%) |

Values are expressed as the number of missing data (percentage of the total number of individuals in the study).

**Supplementary Table 3**. Levels of sNfL and sGFAP of MS group of patients and HC.

| Soluble factors (pg/ml) | HCs (n=180) | NLGL (n=93) | NHGL (n=71) | NHGH (n=55) | NLGH (n=34) |
| --- | --- | --- | --- | --- | --- |
| sNfL | **5.7 (4.4-8.0)** | **6.5 (4.9-7.8)** | **16.9 (131-24.1) ⱡ**** θ**** ¶****** | **21.7 (15.2-43.6) ⱡ**** θ**** ¶****** | 7.4 (6.1-8.8) |
| sNfL Z-score | **-0.4 [-1.2-0.4]** | **0.2 [-0.8-0.8]** | **2.3 [1.8-3.1] ⱡ**** θ**** ¶****** | **2.7 [2.2-3.3] ⱡ**** θ**** ¶****** | 0.6 [-0.1-1.1] |
| sGFAP | **50.2 [62-109]** | **88.8 [65-109.5]** | 100.7 [70.9-123.4] | **191.2 [155-285] ⱡ**** θ**** ¶****** | **181 [159-213] ⱡ**** θ**** ¶****** |

Values are expressed as the median [25-75% IQR] in pg/ml. Significant comparisons are highlighted in bold. Abbreviations: HCs: healthy controls; IQR: interquartile range; NHGH: patients with high sNfL Z-scores and sGFAP values; NHGL: patients with high sNfL Z-scores and normal sGFAP values; NLGH patients with normal sNfL Z-scores and high GFAP levels; NLGL: patients with normal sNfL Z-scores and GFAP values. ⱡ: difference with the HC group; θ: difference with the NLGH group; ¶: difference with the NLGL group; **** p<0.0001 according to Dunn’s test. In all cases, significant p values in Dunn’s test implied p values < 0.05 in the associated Kruskal‒Wallis test.

**Supplementary Table 4**. Serum levels of serum soluble factors of MS group

patients and HC.

| Soluble factors (pg/ml) | HCs (n=180) | NLGL (n=93) | NHGL (n=71) | NHGH (n=55) | NLGH (n=34) |
| --- | --- | --- | --- | --- | --- |
| *Factors associated with inflammation* | | | | | |
| CSF1 | 137 [119-154] | 129 [111-151] | 131 [115-153] | 132 [116-155] | 125 [108-143] |
| CSF3 | 103 [80.8-126] | 96.4 [75.5-144] | 105 [78.7-127] | 100 [85.4-147] | 106 [75.8-144] |
| FLT3LG | **109 [93.4-130]** | 99.4 [78.9-120] | 99.5 [83.4-124] | 102 [81.4-134 | **86.6 [64.7-111] ⱡ***** |
| GM-CSF | **0.15 [0.1-0.2]** | **0.1[0.08-0.2] ⱡ**** | **0.1[0.07-0.15] ⱡ***** | 0.1 [0.08-0.2] | 0.1 [0.06-0.4] |
| IFNG | 0.2 [0.1-0.3] | 0.2 [0.1-0.2] | 0.2 [0.1-0.3] | 0.2 [0.1-0.3] | 0.2 [0.1-0.2] |
| IL1B | 0.2 [0.01-0.6] | 0.2 [0.1-0.4] | 0.2 [0.1-0.5] | 0.2 [0.1-0.3] | 0.2 [0.1-0.4] |
| IL2 | 0.02 [0.01-0.04] | 0.03 [0.02-0.05] | 0.02 [0.01-0.04] | 0.03 [0.02-0.04] | 0.01 [0.02-0.03] |
| IL-4 | **0.05 [0.02-0.08]** | **0.03 [0.02-0.06] ⱡ*** | **0.03 [0.01-0.05] ⱡ*** | **0.02 [0.01-0.06] ⱡ**** | **0.02 [0.01-0.04] ⱡ**** |
| IL6 | 2.2 [1.4-3.4] | 2.4 [1.5-4.7] | 1.8 [1.4-3] | 2.2 [1.5-4.5] | 1.9 [1.3-4] |
| IL7 | 5.5 [4-7.2] | 5.5 [4.1-8] | 5.5 [3.7-7.1] | 4.8 [3.9-7.9] | 5.7 [3.4-7.9] |
| IL10 | 7.7 [5.4-9.7] | 7.4 [4.8-10.3] | 8.1 [5.3-12] | 8.3 [4.9-12.1] | 5.8 [3.9-10.3] |
| IL13 | **0.5 [0.3-1.1]** | **0.3 [0.2-0.7] ⱡ**** | **0.3 [0.2-0.7] ⱡ*** | **0.3 [0.2-0.7] ⱡ**** | **0.2 [0.1-0.8] ⱡ***** |
| IL15 | 12.9 [10.7-15.1 | 12.7 [10.1-15.2] | 12.9 [11.2-15.6] | 12.4 [10.9-15.9] | 12.2 [9.8-16.3] |
| IL17A | 0.4 [0.2-0.7] | 0.4[0.2-0.7] | 0.5[0.2-0.9] | 0.4 [0.2-0.8] | 0.3[0.2-0.9] |
| IL17C | **19.8 [14.7-27.3]** | 18.8 [11.9-29.7] | 19.7 [13.6-26.3] | 18.3 [14.2-27.7] | **15.6 [9.6-22.8] ⱡ*** |
| IL17F | 0.9 [0.5-1.6] | 0.9 [0.5-1.6] | 1.1 [0.6-1.7] | 0.9 [0.4-1.7] | 0.8 [0.4-1.7] |
| IL18 | 279 [228-336] | 270 [211-345] | 268 [217-345] | 261 [220-340] | 241 [200-390] |
| IL27 | 6. [4.7-9.9] | 5.8 [3.9-9.3] | 5.7 [3.8-9.9] | 5.4 [3.4-9.6] | 6.3 [3.7-8] |
| IL33 | 0.1 [0.05-0.2] | 0.1 [0.05-0.2] | 0.1 [0.03-0.2] | 0.1 [0.03-0.2] | 0.1 [0.05-0.2] |
| LTA | 9.4 [7.9-11.2] | 8.4 [6.6-10.9] | 7.9 [6.4-12.2] | 8.3 [6.7-10.3] | 8.3 [6.6-10.8] |
| MMP1 | 3142 [1982-5048] | 2469 [1768-4181] | 2677 [1775-4334] | 2740 [1581-3583] | 1972 [1690-3074] |
| MMP12 | 207 [153-270] | 182 [139-254] | 169 [140-218] | 202 [134-257] | 174 [137-229] |
| OLR1 | 437.9 [236.1-703.9] | 386.9 [273.5-574.7] | 467.6 [3068-732] | 427.6 [271-607] | 329.6 [208.4-546.3] |
| OSM | 9.8 [6.3-19.3] | 10.2 [6.9-16] | 10.3 [6.9-15.9] | 11.4 [6.8-14.5] | 8.5 [5.8-12.4] |
| TNF-α | **19.5 [16.7-23.5]** | 18.4 [15.1-23.2] | 17.6 [14.3-2.8] | 17.3 [13.6-22.7] | **15 [12.7-20.2] ⱡ***** |
| TNFSF10 | 483 [409-581] | 440 [373-515] | 430 [361-509] | 417 [343-520] | 417 [337-516] |
| TNFSF12 | **843 [726-1015]** | 812 [671-960] | 805 [672-951] | 790 [646-945] | **734 [531-855] ⱡ***** |
| TSLP | 0.2 [0.07-0.4] | 0.1 [0.05-0.26] | 0.1 [0.05-0.2] | 0.09 [0.05-0.2] | 0.1 [0.04-0.2] |
| *Chemokines* | | | | | |
| CCL2 | **595 [477-769] θ****** | **532 [397-754] θ**** | **554 [405-714] θ*** | **550 [383-716] θ*** | **397 [209-536]** |
| CCL3 | **9.4 [6.9-22.2]** | 8.3 [5.8-13.1] | 8.5 [5.9-16.1] | 7.6 [5.8-10.3] | **6.2 [5.4-9.7] ⱡ**** |
| CCL4 | **135 [95.3-183]** | **128 [95-182]θ*** | 128 [93.8-170] | 119 [94.4-172] | **99 [70.2-131] ⱡ**** |
| CCL7 | **1.1 [0.8-1.8]** | 0.9 [0.7-1.4] | 0.69 [0.6-1.3] | 0.9 [0.6-1.4] | **0.7 [0.5-1.1] ⱡ**** |
| CCL8 | 61.4 [43.2-81.5] | 58.8 [41.5-85.1] | 60.7 [43.44-81.7] | 63.2 [48.1-82.8] | 50.9 [32.4-75.6] |
| CCL11 | **154 [109-194]** | 123 [102-172] | 125 [95.2-171] | 116 [100-171] | **113 [78.2-151] ⱡ*** |
| CCL13 | **164 [117-223]** | 151 [101-235] | 141 [106-209] | 149 [92.1-211] | **81.8 [120-176] ⱡ*** |
| CCL19 | 96.4 [78.7-138.6] | 90.9 [70.1-138] | 89.9 [68.4-151] | 99.9 [70.6-153] | 90.3 [69.7-121] |
| CXCL8 | **21.2 [14.2-112]** | 17.1 [12-28.9] | 21.1 [13.1-59.7] | 15.9 [13.1-24.6] | **10.1 [12.8-23.7] ⱡ*** |
| CXCL9 | 46.1 [35.7-73.6] | 42.6 [33.7-65.1] | 41.3 [29.3-59] | 40.4 [28.8-71.3] | 40.8 [32.3-65.1] |
| CXCL10 | 85.7 [63.7-125] | 83.1 [59.5-120] | 78.4 [59.3-117] | 73.9 [58.6-118] | 67.2 [46.3-127] |
| CXCL11 | 64.3 [47.3-88.8] | 63.5 [43.1-94.3] | 55.9 [37.9-78.9] | 56.3 [37.3-75.6] | 49.5 [37.2-103] |
| CXCL12 | 197.6 [161.9-233.2] | 193.5 [158.9-236.5] | 195.5 [169-227.3] | 209 [155-245] | 192.4 [164.6-219.8] |
| *Factors involved in repair processes* | | | | | |
| EGF | **604 [359-750]** | **383[209-614] ⱡ**** | **512 [304-727]** | **343 [165-539] ⱡ**** ϒ*** | **311 [127-182] ⱡ**** ϒ**** |
| HGF | 589 [451-817] | 557 [445-761] | 553 [426-779] | 584 [483-774] | 510 [410-680] |
| TGFA | 22.6 [14.8-31.7] | 24.3 [17.5-33.8] | 24.5 [18-33.2] | 26.1 [20-37.9] | 21.8 [14.4-35.5] |
| VEGFA | **704 [454-981]** | 612 [467-938] | 570 [410-812] | 575 [387-740] | **460 [345-778] ⱡ*** |

Values are expressed as the median [25-75% IQR] in pg/ml. Significant comparisons are highlighted in bold. Abbreviations: HCs: healthy controls; IQR: interquartile range; NHGH: patients with high sNfL Z-scores and sGFAP values; NHGL: patients with high sNfL Z-scores and normal sGFAP values; NLGH patients with normal sNfL Z-scores and high GFAP levels; NLGL: patients with normal sNfL Z-scores and GFAP values. ⱡ: difference with the HC group; θ: difference with the NLGH group; ϒ: difference with the NHGL group; * p<0.05, ** p<0.01, *** p<0.001, and **** p<0.0001 according to Dunn’s test. In all cases, significant p values in Dunn’s test implied p values < 0.05 in the associated Kruskal‒Wallis test.


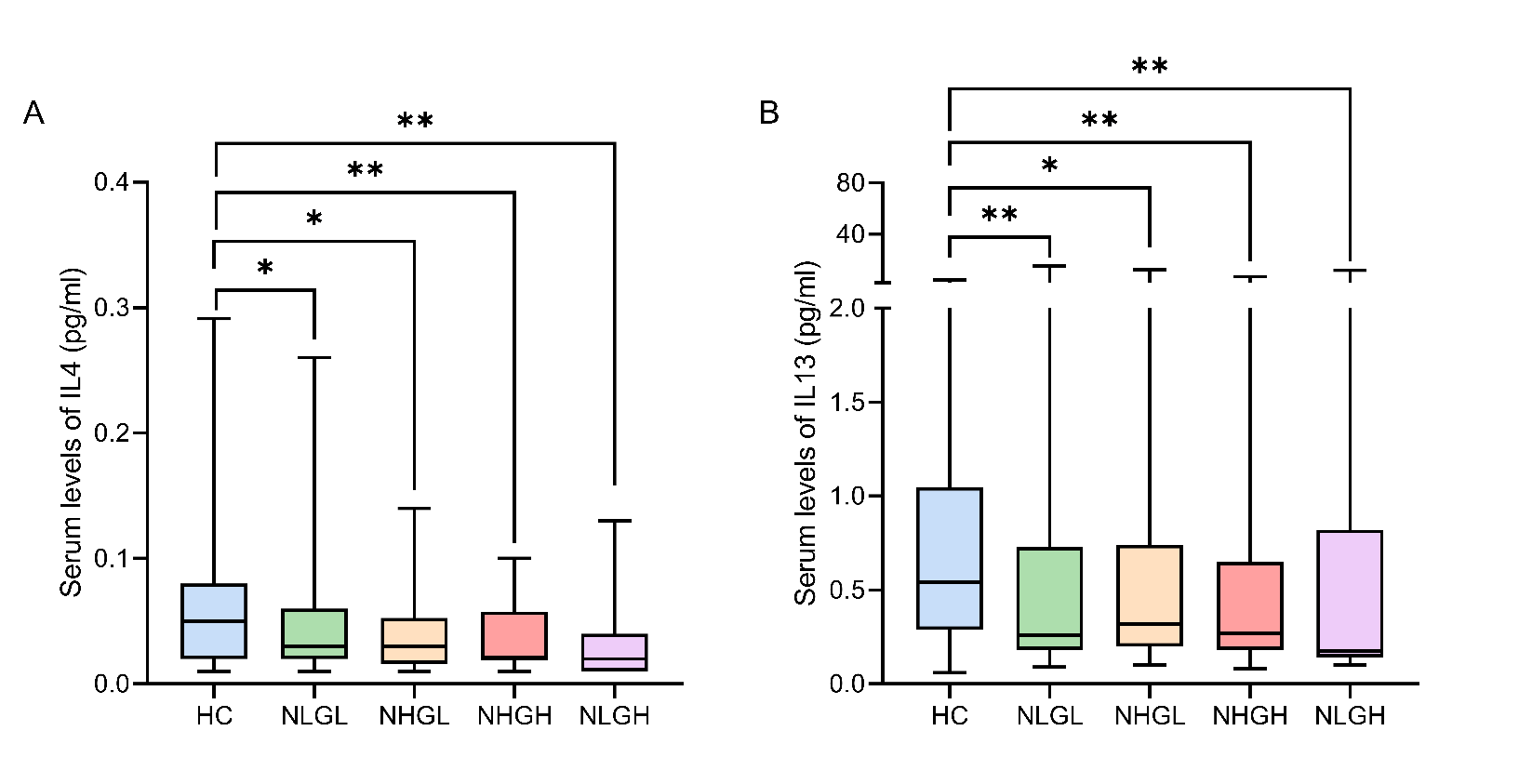


Supplementary Figure 1. Serum levels (pg/mL) of Th2 cytokines in PwMS and HCs: IL13 (A) and IL4 (B). Abbreviations: HCs: Healthy controls; IL4: Interleukin-4; IL13: Interleukin-13 NHGL: Patients with high sNfL Z-scores and normal sGFAP values; NLGH: Patients with low sNfL Z-scores and high GFAP levels; NLGL: Patients with low sNfL Z-scores and GFAP values; PwMS: patients with multiple sclerosis; * q<0.05; and ** q<0.01 obtained from Dunn’s post-hoc test and adjusted using the false discovery rate method.
